# Real-world outcomes of tumour necrosis factor inhibitor (TNFi) cyclers versus mechanism-of-action switchers among ulcerative colitis patients who failed first-line TNFi

**DOI:** 10.1186/s12876-026-04829-y

**Published:** 2026-06-20

**Authors:** Navneet Upadhyay, Amita Ketkar, Can Mert, Alexandra Wallem, Simin K. Baygani, Tariku J. Beyene, Deborah A. Fisher, Tracey Quimbo, Raven A. Perez, Michael Grabner, J. Casey Chapman

**Affiliations:** 1 Eli Lilly and Company, 893 Delaware St, Indianapolis, IN 46225 USA; 2 Carelon Research, Wilmington, DE USA; 3 HaaPACS GmbH, Schriesheim, Germany; 4 GI Alliance, Southlake, TX USA

**Keywords:** Ulcerative colitis, Tumour necrosis factor inhibitor, Real-world effectiveness

## Abstract

**Background:**

We compared real-world outcomes among patients with ulcerative colitis (UC) prescribed second-line tumour necrosis factor inhibitor (TNFi) versus non-TNFi therapy after a first-line TNFi.

**Methods:**

We included adults with ≥ 2 claims for UC and ≥ 1 medical or pharmacy claim for a UC-approved TNFi as first-line therapy between 1 January 2015 and 30 June 2022 from the Healthcare Integrated Research Database. Second-line therapy was identified by one or more claims for a different TNFi or non-TNFi. The primary outcome was inadequate response, a composite of at least one of switch/add alternative advanced therapy, augment with conventional therapy, dose escalation, glucocorticoid intensification, UC-related hospitalisation, or UC-related surgery. We used multivariable logistic regression with trimmed inverse probability treatment weighting (IPTW) to calculate adjusted odds of inadequate response with second-line TNFi versus non-TNFi.

**Results:**

Among 921 IPTW-weighted patients (second-line TNFi, *n* = 281; non-TNFi, *n* = 640), 62% of the TNFi cohort versus 49% non-TNFi had inadequate response (chi-square *p* = 0.001; odds ratio [95% CI], 1.76 [1.31–2.37], *p* < 0.001). Mean/median time to inadequate response among patients with inadequate response within 1 year was 139/125 days (TNFi) and 150/130 days (non-TNFi). The most common criterion for inadequate response was addition of new conventional therapy (35% second-line TNFi vs. 25% non-TNFi, *p* < 0.01); switch to or addition of alternative advanced therapy (30% vs. 15%, *p* < 0.01), and dose increase of oral glucocorticoids (14% vs. 9%, *p* < 0.05) were also significantly more common among patients in the TNFi cohort than the non-TNFi cohort. Total healthcare costs were modestly higher in the TNFi cohort.

**Conclusions:**

These results suggest that patients with UC have higher odds of inadequate response with a second TNFi versus non-TNFi after a first TNFi failure. Switching to an alternate mechanism of action for second-line therapy yielded better clinical outcomes.

**Supplementary Information:**

The online version contains supplementary material available at https://doi.org/10.1186/s12876-026-04829-y.

## Background

Ulcerative colitis (UC) is a chronic inflammatory bowel disease with an estimated prevalence in the United States (US) of 181.1 per 100,000 people [1]. Common symptoms include bloody diarrhoea, abdominal pain, rectal bleeding, bowel urgency and incontinence, weight loss, and fatigue [2]. Around one in five patients with UC experience disease-related hospitalisation within 5 years of diagnosis [3], and approximately 10% undergo colectomy during the 10 years after diagnosis [4]. The economic burden of UC is estimated at $15-$32 billion per year [5].

For moderate-to-severe UC, treatment guidelines recommend advanced therapies; that is, biologics and small molecules [6, 7]. Tumour necrosis factor inhibitors (TNFis) have been the predominant first-line advanced therapies [8, 9]; however, up to 30% of patients with inflammatory bowel disease lack a response to initial TNFi treatment (primary nonresponse), and a similar or greater proportion lose response over time (secondary nonresponse) or discontinue therapy because of intolerance [10, 11]. In two real-world studies of patients with UC, more than half of the patients experienced nonresponse to first-line TNFi therapy [9, 12].

Following the failure of first-line TNFi therapy, patients may switch to a different TNFi or to another class of advanced therapy. Both the American College of Gastroenterology (ACG) and American Gastroenterological Association (AGA) guidelines recognise the benefit of switching to a drug with an alternative mechanism of action for patients who do not achieve a therapeutic benefit with TNFi therapy despite adequate drug concentrations [6, 7].

However, limited data are available on real-world practice patterns and outcomes with TNFis compared with non-TNFis as second-line therapy in UC. These data are important to inform healthcare decision-making and identify unmet needs for patients, practitioners, and payers. The few studies that have evaluated real-world treatment patterns and healthcare resource utilisation (HCRU) for second-line TNFi versus non-TNFi therapy have been limited to specific agents or treatment sequences [13–16] or were conducted in a broader population of patients with chronic inflammatory conditions [17]. For example, a retrospective study by Hupé et al. showed that UC patients who failed a first subcutaneous TNFi agent were more likely to achieve clinical remission with vedolizumab than those treated with infliximab [18]. 

To our knowledge, no published studies to date have evaluated second-line outcomes across therapeutic classes specifically among patients with moderate-to-severe UC. In one real-world study presented at a major medical conference, patients with UC who were switched to a non-TNFi after initial TNFi treatment experienced higher persistence rates and a longer time to treatment discontinuation than patients receiving a second TNFi [19].

This study compared treatment response, persistence, and HCRU among patients with moderate-to-severe UC in the US who received second-line TNFi or non-TNFi therapy after failure of a first-line TNFi, using a claims-based algorithm adapted from prior literature [9, 20].

## Methods

### Study design

This was a retrospective, observational cohort study using the Healthcare Integrated Research Database (HIRD^®^), a large administrative healthcare database maintained by Carelon Research containing medical claims (including inpatient, emergency department [ED], and all outpatient locations), pharmacy claims, and other types of information. This study used US commercial, Medicare Advantage, and Medicare Supplement/Part D longitudinal pharmacy and medical claims. Additional information and technical details about HIRD data contents, quality, provenance, and representativeness have been previously published [21].

The HIRD is a limited dataset, and data use agreements with covered entities were in place in accordance with the Health Insurance Portability and Accountability Act Privacy Rule; hence, Institutional Review Board approval was not required.

The study protocol was finalised prior to accessing the data source.

The study period was 1 January 2014 to 30 June 2023, and the patient identification (first-line therapy intake) period was 1 January 2015 to 30 June 2022 (Supplementary Fig. 1).

### Study population and definitions

Patient selection criteria are detailed in Supplementary Table 1. Inclusion criteria were having two or more claims for UC (at least 30 days apart) during the study period; a medical or pharmacy claim for a TNFi or non-TNFi advanced therapy approved for the treatment of UC during the patient identification period (with the earliest claim set as the index date) following a TNFi first-line agent (with the earliest claim in this category set as the first-line claim date); age 18 years or older on the index date; and continuous medical and pharmacy enrolment from 1 year before the first-line TNFi claim date to 1 year after the index date (patients who disenrolled or switched to health plans not tracked in the HIRD were excluded). Therapies approved in the US for the treatment of UC during the patient identification period were adalimumab, golimumab, and infliximab (TNFi therapies) and ozanimod, tofacitinib, upadacitinib, ustekinumab, and vedolizumab (non-TNFi advanced therapies).

Patients were excluded if they had more than one TNFi approved for UC on the first-line claim date; any claims for a TNFi or non-TNFi over 1 year prior to the first-line claim date (this constituted a “washout period” to better identify true first-line TNFi users); more than one TNFi or non-TNFi advanced agent approved for UC on the index date; a total colectomy; a diagnosis of Crohn’s disease (CD); or two or more claims 30 days apart for non-UC autoimmune conditions during the baseline period.

The baseline (pre-index) period covered 183 days before the index date (first 2 L claim) to 1 day prior to the index date. The follow-up period covered the index date and the following 365 days (inclusive).

In the claims data, medications were identified via Healthcare Common Procedure Coding System codes, procedures via Current Procedural Terminology codes, and medical conditions via International Classification of Diseases (ICD)-9-Clinical Modification (CM) and ICD-10-CM diagnosis codes.

### Outcome measures

#### Inadequate response

The primary endpoint was inadequate response, which was assessed during the 1-year follow-up period for patients who received TNFi or non-TNFi second-line therapy. The claims-based inadequate response algorithm was developed and validated for rheumatoid arthritis [20] and was previously modified for the UC setting [9]. The algorithm was further modified for this study based on clinical input. A patient was classified as having an inadequate response if any one of the following individual criteria was met: switch to or addition of alternative advanced therapy, augmentation with conventional therapy, dose escalation of the index medication, increase in the dose of oral glucocorticoids, use of intravenous glucocorticoids, UC-related hospitalisation or ED visit, or UC-related surgery. The dose escalation variable identified post-induction instances where a dose was at least twice the labeled dose. This high threshold was chosen to avoid incorrectly flagging dose adjustments due to individual response heterogeneity.

Additional details of the inadequate response algorithm are presented in the Supplementary Methods: Inadequate response Individual criteria.

#### Persistence/adherence

The secondary endpoint was persistence during the 1-year follow-up period for patients who received TNFi or non-TNFi second-line therapy. Persistent days were defined as the number of days of continuous therapy from initiation (index date) until the end of the 1-year follow-up period, allowing for a maximum fixed gap of 45 days between fill date plus days’ supply (for Rx claims) or dosage frequency (for medical claims) and the subsequent fill date (days’ supply was imputed as necessary; see Supplementary Methods: Calculation of proportion of days covered [PDC]). If the number of persistent days equalled or exceeded the number of days in the 1-year follow-up period, the patient was considered persistent. Otherwise, the patient was considered non-persistent (discontinued). Date of treatment discontinuation was defined as the last fill date in the continuous treatment segment plus days’ supply of that fill. Persistence is reported as the proportion of patients who were persistent throughout 1 year of follow-up. We also report time to discontinuation among those who discontinued.

Adherence was defined based on PDC < 80%. PDC was calculated as the ratio of the total number of days with the drug on hand to the length of the follow-up period (i.e., 365 days; see Supplementary Methods: Calculation of PDC for additional details).

#### Other measures

Other measures included demographic characteristics (assessed at the index date) and clinical characteristics during the baseline period (also referred to as baseline characteristics). Comorbidities were recorded based on the Quan-Charlson Comorbidity Index (QCI) [22] and additional targeted comorbidities known to be important in this patient population. Conventional and advanced therapy use (both first and second-line) is also reported. Conventional therapies included aminosalicylates, corticosteroids, immunomodulators, methotrexate, and leflunomide.

All-cause HCRU and costs, as well as UC-related HCRU and costs (defined as a UC code found in any position on the medical claim line or presence of conventional/advanced therapies for UC), were assessed over the 1-year follow-up period. HCRU included inpatient admissions, ED visits, and outpatient visits (all from medical claims) and pharmacy fills (from pharmacy claims).

Costs represented the combined total amount paid by the health plan, the patient, and third parties. Costs for inpatient, ED, and outpatient visits were captured. In reporting UC-related costs, conventional and advanced UC therapy costs covered under the medical and pharmacy benefits were recorded. Costs were adjusted to 2022 US dollars based on the most recent medical care price index information provided by the US Bureau of Labour Statistics.

### Statistical analyses

Descriptive statistics, including means and standard deviations and/or medians and interquartile ranges for continuous data, and frequencies and proportions for categorical data, are reported separately for the TNFi and non-TNFi cohorts.

Comparisons between the TNFi and non-TNFi cohorts assumed an intention-to-treat/ “as started” approach. To reduce potential bias from measured confounders, cohorts of patients receiving TNFi and non-TNFi therapies were balanced for pre-treatment variables using inverse probability treatment weighting (IPTW). IPTW involved the following steps: (1) propensity scores predicting the likelihood of receiving TNFi or non-TNFi treatment were computed using binomial logistic regression, taking into account potential confounders; (2) propensity scores were transformed into weights, and balance was assessed using standardised mean differences (SMDs; means were considered balanced if SMD < 0.1 in absolute value) and variance ratios (with variance considered balanced if in the range of 0.5–2); (3) weights were adjusted using stabilisation and trimming (at the 1st and 99th percentiles), cost variables were log-transformed for balance assessment, and weighted cohorts were evaluated before between-cohort outcomes analyses were conducted. Imbalanced variables post-weighting were included as covariates in the multivariable regression models. Further details can be found in Supplementary Methods: Detailed explanation of IPTW.

For descriptive purposes, statistical comparisons of treatment outcomes were performed between the IPTW-weighted TNFi and non-TNFi cohorts using weighted two-tailed *t*-tests for continuous variables and chi-square tests for categorical variables, with *p*-values < 0.05 indicating statistical significance. We also report SMDs as a measure of effect size (with SMD ≥ 0.1 indicating the presence of differences).

The main regression analysis for the primary outcome of the inadequate response composite endpoint was performed via multivariable logistic regression using IPTW as weights; all baseline-imbalanced variables were included in this regression in a doubly robust approach [23].

To assess the robustness of the main regression analysis, three sensitivity analyses were performed. The first sensitivity analysis consisted of a regression model with IPTW, a selection of baseline-imbalanced variables, and all variables used for generating the propensity scores. The second sensitivity analysis consisted of a regression model with IPTW, all baseline-imbalanced variables, and all variables used for generating the propensity scores. The third sensitivity analysis was a weighted regression model without covariates. Covariates for each regression model are listed in Supplementary Table 2.

For persistent days (time until end of follow-up or index drug discontinuation), time to the inadequate response composite endpoint, and time to each individual inadequate response criterion, multivariable adjusted Cox proportional hazards regression analyses using the IPTW-weighted cohorts were used to estimate hazard ratios (HRs) and construct adjusted survival curves. *P*-values were calculated for censored log-rank analyses. Due to insufficient patient counts, time-to-event analyses were not performed for two of the inadequate response criteria (use of intravenous glucocorticoids and UC-related surgery).

HCRU and costs for the TNFi and non-TNFi cohorts were compared using IPTW-adjusted Chi^2^ tests as well as generalised linear models with log link and negative binomial or Gamma distributions.

All analysis was conducted using a combination of the Instant Health Data (IHD) platform (Panalgo, Boston, MA), R, version 4.0.2 (R Foundation for Statistical Computing, Vienna, Austria), and Statistical Analysis Software (SAS) Enterprise Guide, version 9.4 (SAS Institute Inc., Cary, NC, USA). An alpha level of 0.05 was used to identify statistical significance and no adjustments were made for multiple comparisons.

## Results

### Patient selection

**Fig. 1:**
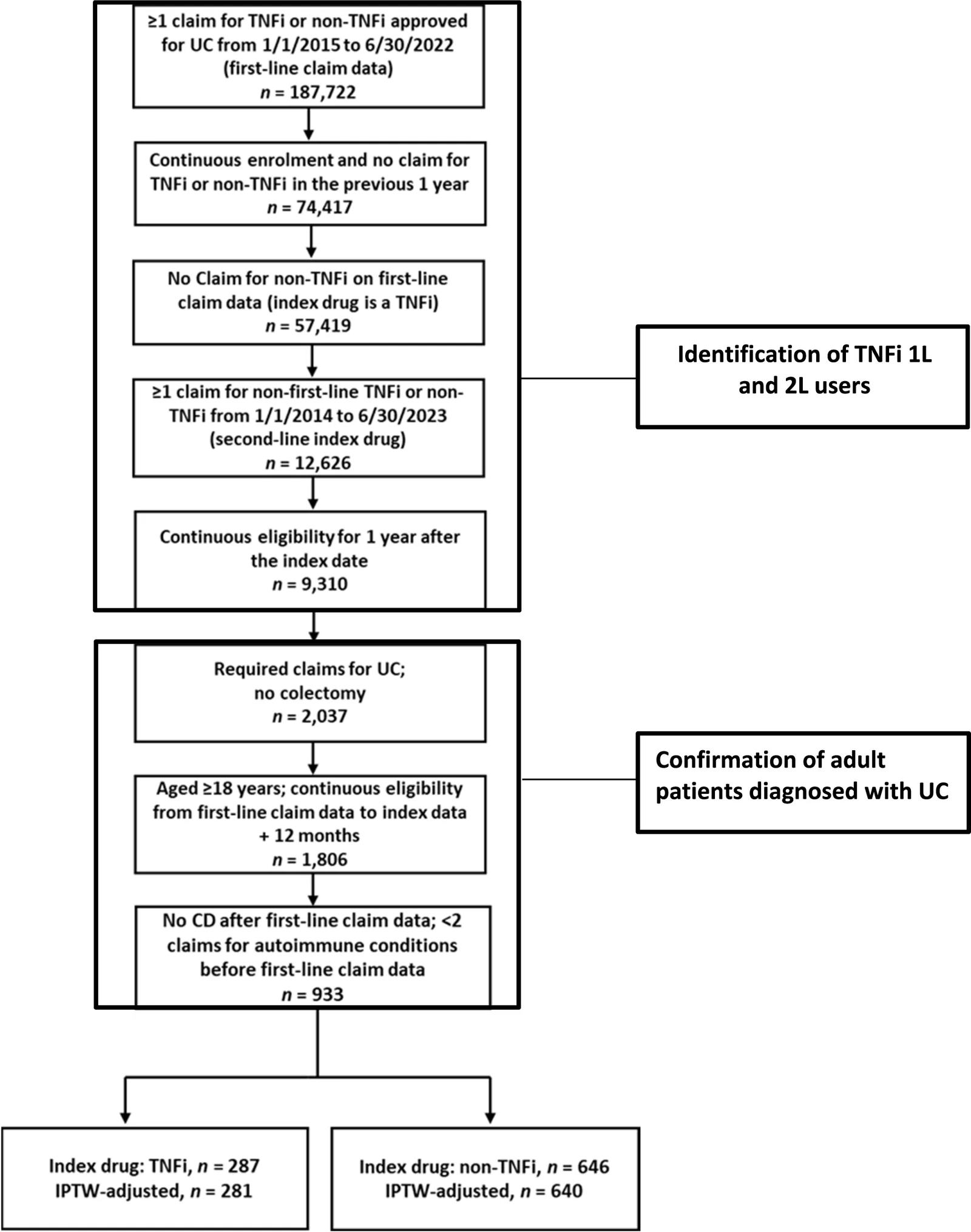
Flow diagram of patient selection. See Supplementary Table 1 for additional details. Abbreviations: CD, Crohn’s disease; IPTW, inverse probability treatment weighting; TNFi, tumour necrosis factor inhibitor; UC, ulcerative colitis

A total of 933 patients met all inclusion and exclusion criteria, of whom 287 received a TNFi and 646 received a non-TNFi advanced agent as second-line (index) therapy (Fig. 1 and Supplementary Table 1). The weighted IPTW analysis included 921 patients, of whom 281 (31%) were prescribed a TNFi and 640 (69%) a non-TNFi.

### Patient characteristics

The demographic and clinical characteristics of patients receiving TNFi or non-TNFi second-line therapy were generally balanced in the IPTW-weighted samples (Table 1). Patients in each cohort had a mean age of approximately 43 years, 56% were female, and most were enrolled in preferred provider organisation plans. In each cohort, the mean QCI was 0.4, and more than 80% of patients had a QCI of zero. Common comorbidities not measured in the QCI included infection, anaemia, hypertension, dyslipidemia, and anxiety (Table 1).

**Table 1:** Demographic and clinical characteristics in the IPTW-weighted sample

	TNFi (*n* = 281)	Non-TNFi (*n* = 640)	SMD
Age, years			
Mean (SD)	42.5 (14.2)	42.7 (15.0)	0.01
Median (IQR)	42 (32–53)	42 (31–54)	
Age categories, *n* (%)			
18–39	122 (44)	277 (43)	0.00
40–64	143 (51)	317 (50)	0.03
65–74	9 (3)	35 (6)	0.11
≥ 75	6 (2)	11 (2)	0.04
Female sex, *n* (%)	124 (44)	279 (44)	0.01
Region of residence, *n* (%)			
Northeast	44 (16)	99 (15)	0.01
Midwest	49 (18)	129 (20)	0.07
South	121 (43)	258 (40)	0.05
West	61 (22)	120 (19)	0.07
Other/unknown	6 (2)	34 (5)	0.16
Health plan type, *n* (%)			
CDHP	52 (18)	131 (21)	0.05
HMO	53 (19)	111 (17)	0.04
PPO	177 (63)	398 (62)	0.02
Index year, *n* (%)			
2015	7 (3)	15 (2)	0.01
2016	32 (11)	68 (11)	0.03
2017	42 (15)	98 (15)	0.01
2018	41 (15)	90 (14)	0.01
2019	37 (13)	90 (14)	0.03
2020	42 (15)	97 (15)	0.00
2021	43 (15)	102 (16)	0.02
2022	37 (13)	80 (13)	0.02
QCI, mean (SD)	0.4 (1.2)	0.4 (0.9)	0.03
QCI category, *n* (%)			
0	232 (83)	520 (81)	0.04
1	23 (8)	49 (8)	0.02
2	11 (4)	51 (8)	0.16
≥ 3	14 (5)	20 (3)	0.09
Targeted comorbidities, *n* (%)			
Anaemia	88 (31)	175 (27)	0.09
Dyslipidemia	46 (16)	94 (15)	0.05
Fibromyalgia	< 5 (< 2)	< 5 (< 1)	0.04
Hypertension	54 (19)	122 (19)	0.00
Infection	114 (41)	262 (41)	0.01
Anxiety	43 (16)	96 (15)	0.01
Depression	23 (8)	51 (8)	0.00
Obesity	25 (9)	67 (11)	0.05

IPTW-weighted cohort. Values from 1 to 4 are replaced with “<5” to protect patient privacy

*Abbreviations*: *CDHP* Consumer-driven health products, *HMO* Health maintenance organisation, *IPTW* Inverse probability treatment weighting, *IQR* Interquartile range, *PPO* Preferred provider organisation, *QCI* Quan-Charlson Comorbidity Index, *SD* Standard deviation, *SMD* Standardised mean difference (balanced if < 0.1), *TNFi* tumour necrosis factor inhibitor

Infliximab, followed by adalimumab, was the TNFi most prescribed as second-line therapy (Table 2). Vedolizumab was the most common second-line non-TNFi advanced treatment, followed by ustekinumab and tofacitinib. The use of conventional therapies at baseline was similar between the two cohorts (Table 2). Adalimumab was the most prescribed TNFi as first-line therapy, and the mean time on first-line treatment was slightly more than 1 year (Table 2).

**Table 2:** Treatment patterns at baseline or on index date

	TNFi (*n* = 281)	Non-TNFi (*n* = 640)	SMD
First-line advanced therapy			
TNFi, *n* (%)			
Adalimumab	171 (61)	398 (62)	0.02
Golimumab	14 (5)	30 (5)	0.02
Infliximab	95 (34)	213 (33)	0.02
Time on therapy*, days, mean (SD)	371 (373.3)	380 (355.3)	0.02
Time between last fill and index date, days, mean (SD)	119 (228)	100 (176)	0.09
Second-line advanced therapy, *n* (%)			
TNFi			
Adalimumab	102 (36)	0	N/A
Golimumab	42 (15)	0	N/A
Infliximab	137 (49)	0	N/A
Non-TNFi			
Ozanimod	0	< 5 (1)	N/A
Tofacitinib	0	63 (10)	N/A
Upadacitinib	0	< 5 (1)	N/A
Ustekinumab	0	91 (14)	N/A
Vedolizumab	0	474 (74)	N/A
Conventional therapy at baseline, *n* (%)			
Aminosalicylate	149 (53)	362 (57)	0.07
Corticosteroid	210 (75)	494 (77)	0.06
Immunomodulator	56 (20)	124 (19)	0.01
Other conventional therapy	9 (3)	18 (3)	0.02

IPTW-weighted cohort. Values from 1 to 4 are replaced with “<5” to protect patient privacy

*Abbreviations*: *IPTW* Inverse probability treatment weighting, *N/A* Not applicable, *SMD* Standardised mean difference (balanced if < 0.1), *TNFi* Tumour necrosis factor inhibitor

*Time between first claim for 1 L therapy and first claim of 2 L therapy (i.e., index date)

### Inadequate response

In the IPTW-weighted cohorts, a significantly larger proportion of patients prescribed a TNFi as second-line therapy experienced inadequate response compared with patients receiving a non-TNFi (62% vs. 49%; *p* *<* 0.001) during the 12 months after the index date (Table 3).

**Table 3:** Treatment outcomes over 12 months after the index date

	TNFi (*n* = 281)	Non-TNFi (*n* = 640)	*p*-value	SMD	Odds/Hazard ratio estimate (95% CI)^a^	*p*-value^b^
Inadequate response (composite endpoint), *n* (%)	174 (62)	316 (49)	0.001	0.25	1.76 (1.31–2.37)	< 0.001
Inadequate response, individual criteria, *n* (%)					-	-
Switch to or addition of alternative advanced therapy	85 (30)	95 (15)	< 0.001	0.37	2.35 (1.75–3.17)	< 0.001
Addition of a new conventional therapy	98 (35)	161 (25)	0.003	0.21	1.53 (1.18–1.97)	0.001
Dose escalation of index medication	41 (14)	79 (12)	0.373	0.06	1.21 (0.83–1.78)	0.328
Increase in dose of oral glucocorticoids	40 (14)	56 (9)	0.011	0.18	1.67 (1.11–2.51)	0.014
Use of IV glucocorticoids^c^	7 (2)	15 (2)	0.947	0.00	-	-
UC-related hospitalisation or ED visit	47 (17)	91 (14)	0.355	0.07	1.33 (0.92–1.91)	0.128
UC-related surgery^c^	10 (4)	34 (5)	0.236	0.09	-	-
Number of criteria satisfied for inadequate response, mean (SD)	1.2 (1.2)	0.8 (1.1)	< 0.001	0.30	-	-
Time to inadequate response, days^d^					-	-
Mean (SD)	139 (95.2)	150 (101.1)	0.221	0.11	-	-
Median (IQR)	125 (63–218)	130 (74–222)			-	-
Persistent on index therapy at 12 months, *n* (%)	142 (51)	392 (61)	0.003	0.22	-	-
Time to index drug discontinuation, days^e^					1.43 (1.16–1.77)	0.001
Mean (SD)	171 (108.1)	162 (104.9)	< 0.001	0.12	-	-
Median (IQR)	155 (75–259)	153 (67–259)			-	-
Low adherence (PDC < 80%), *n* (%)	122 (43)	205 (32)	0.001	0.23	-	-
PDC					-	-
Mean (SD)	0.7 (0.3)	0.8 (0.3)	< 0.001	0.29	-	-
Median (IQR)	0.9 (0.4–1)	0.9 (0.7–1)			-	-

IPTW-weighted cohort. *Abbreviations*: *CI* Confidence interval, *ED* Emergency department, *IPTW* Inverse probability of treatment weighting, *IQR* Interquartile range, *IV* Intravenous, *PDC* Proportion of days covered, *QCI* Quan-Charlson Comorbidity Index, *SD* Standard deviation, *SMD* standardised mean difference (effect present if ≥ 0.1), *TNFi* Tumour necrosis factor inhibitor; UC, ulcerative colitis

^a^Odds ratio is presented for composite (overall) inadequate response, and hazard ratios are presented for all other analyses

^b^*p*-value calculated from logistic regression model for composite (overall) inadequate response and from Cox proportional hazards regression models for all other analyses. Covariates included in the primary regression model for inadequate response are reported in Supplementary Table 2. Each of the Cox proportional hazards models included the following covariates: cohort, age, sex, UC-related length of stay, region (missing vs. nonmissing), QCI score, QCI comorbidities (diabetes without complication, diabetes with complication, metastatic solid tumour), and baseline conditions (Crohn’s disease, spondylitis, pyoderma gangrenosum, autoimmune hepatitis, mesalamine use)

^c^Cox proportional hazards regression model could not be executed due to insufficient patient counts

^d^Calculated only among those with inadequate response

^e^Calculated only among those who discontinued the index drug

Based on the IPTW-weighted multivariable logistic regression model, patients receiving a TNFi as second-line therapy had 76% higher odds of having an inadequate response than those receiving non-TNFi therapy (odds ratio [OR], 1.76; 95% confidence interval [CI], 1.31–2.37; *p* < 0.001; see Table 3 and Supplementary Table 2 for the list of variables included in the regression model). Sensitivity analysis of the IPTW-weighted multivariable regression model with and without baseline variables resulted in similar ORs (Supplementary Table 3).

The most common individual criterion for inadequate response was addition of a new conventional therapy (35% for second-line TNFi; 25% for non-TNFi; Table 3). Switch to or addition of alternative advanced therapy, addition of a new conventional therapy, and dose increase of oral glucocorticoids were significantly more common among patients in the TNFi cohort than the non-TNFi cohort. Based on IPTW-weighted Cox proportional hazards regression analyses, patients receiving TNFi had a significantly greater hazard of experiencing each of these three events than patients who received non-TNFi second-line therapy (Table 3).

**Fig. 2:**
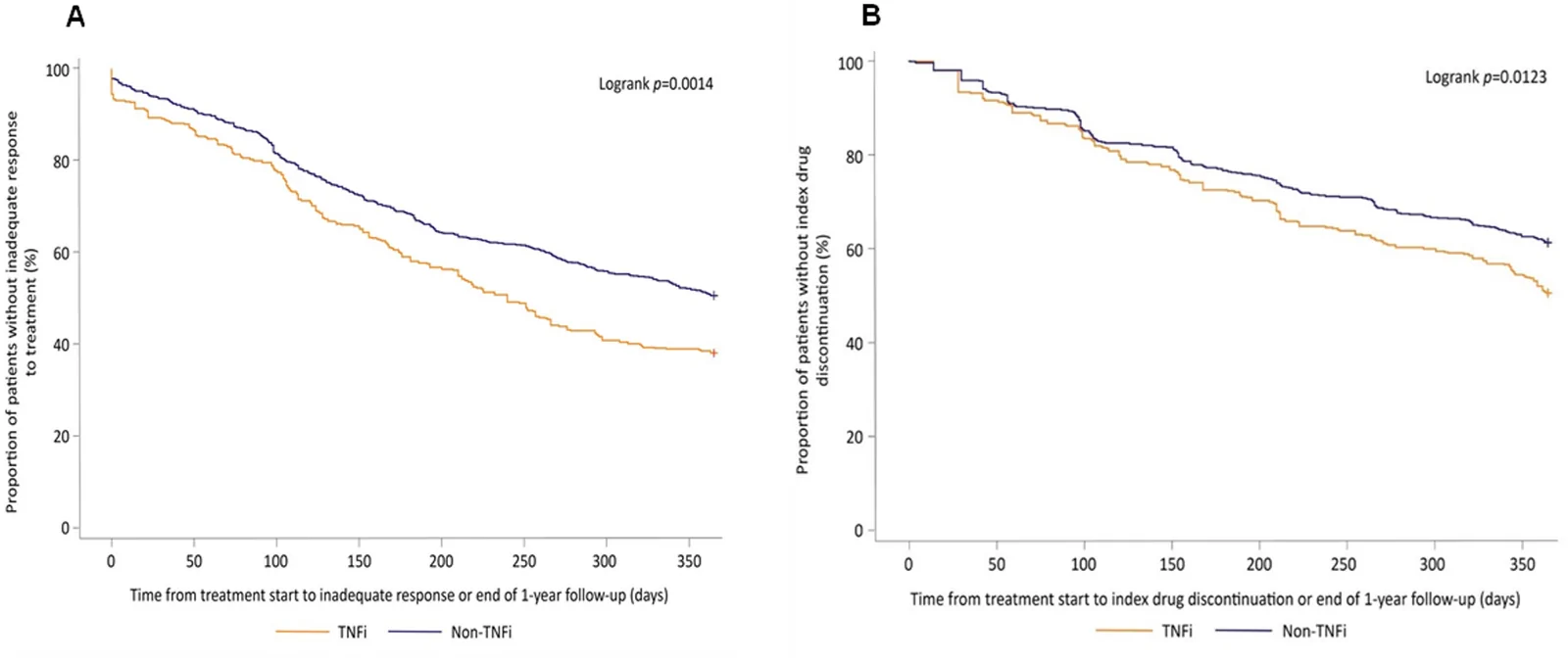
Adjusted survival curves for time to (**A**) the inadequate response composite endpoint (adjusted HR of 1.48 [95% CI, 1.23–1.79]; *p* < 0.001, with non-TNFi as reference) and (**B**) index drug discontinuation over the 12 months after the index date (adjusted HR of 1.43 [95% CI, 1.16–1.77]; *p* < 0.001, with non-TNFi as reference). The survival curves are weighted and adjusted for the covariates in the respective Cox regression models (see Table 3). Abbreviations: +, censored; CI, confidence interval; HR, hazard ratio; TNFi, tumour necrosis factor inhibitor

The mean/median time to inadequate response among patients with an inadequate response within the first year was similar between TNFi (139/125 days) and non-TNFi (150/130 days) users (Table 3). Time to inadequate response estimated via survival analysis is illustrated in Fig. 2A (*p =* 0.0014).

### Treatment persistence and adherence

A greater proportion of patients prescribed non-TNFi second-line treatment persisted on therapy throughout the first year of follow-up compared with patients prescribed TNFi (61% vs. 51%, *p* = 0.003; Table 3). The mean time to index drug discontinuation (among those who discontinued) was slightly longer for patients receiving TNFi than non-TNFi treatment (171 vs. 162 days, *p* < 0.001; Table 3). Time to index drug discontinuation (persistent days) estimated via survival analysis is illustrated in Fig. 2B (*p* = 0.012); the adjusted HR was 1.43 (95% CI, 1.16–1.77; *p <* 0.001).

Low adherence (PDC < 80%) was observed in more patients prescribed TNFi versus non-TNFi (43% vs. 32%; *p* = 0.001), and mean PDC was significantly lower in the TNFi cohort (Table 3).

### Healthcare resource utilisation and costs

Total healthcare costs were modestly higher in the TNFi cohort ($114,608 vs. $99,188, *p* = 0.002), driven by higher inpatient and pharmacy costs, partially offset by lower outpatient costs (Table 4). The pattern of findings was similar for UC-related costs (Supplementary Table 4). UC-related therapy costs constituted a large proportion of total UC-related costs, and costs associated with UC represented most of the all-cause costs in this patient population (Table 4 and Supplementary Table 4).

**Table 4:** All-cause HCRU and costs over 12 months after the index date

All-cause	TNFi (*n* = 281)	Non-TNFi (*n* = 640)	*p*-value	SMD
HCRU				
Inpatient				
≥ 1 visit, *n* (%)	54 (19)	99 (15)	0.155	0.10
Visits, mean (SD)	0.4 (1)	0.3 (1)	0.174	0.11
Length of stay among those with ≥ 1 visit, days, mean (SD)	7.5 (10.4)	7 (7.1)	0.563	0.06
ED				
≥ 1 visit, *n* (%)	60 (21)	126 (20)	0.571	0.04
Visits, mean (SD)	0.3 (1)	0.3 (1)	0.802	0.03
Outpatient				
≥ 1 visit, *n* (%)	280 (100)	639 (100)	0.444	0.05
Visits, mean (SD)	33.6 (23.6)	32.4 (22)	0.693	0.05
Pharmacy fills, mean (SD)	23.5 (14.6)	19.3 (13.9)	< 0.001	0.30
Costs, $, mean (SD) [median]^a^				
Total medical	66,071 (163,236) [28,679]	62,537 (69,968) [56,329]	0.493	0.03
Inpatient	23,605 (146,846) [0]	8,382 (29,278) [0]	< 0.001^b^	0.14
ED	1,467 (4,608) [0]	1,165 (3,505) [0]	0.315^b^	0.07
Outpatient	40,999 (61,713) [22,480]	52,990 (60,269) [52,047]	< 0.001	0.20
Pharmacy	48,537 (45,942) [37,141]	36,651 (55,036) [9,057]	0.014	0.23
Total	114,608 (159,947) [93,516]	99,188 (76,055) [77,305]	0.002	0.12

IPTW-weighted cohort. The mean numbers of visits/fills and mean costs are calculated out of the total number of patients in each cohort. *p*-values were calculated as follows: Chi^2^ tests for HCRU percentage comparisons; GLM with negative binomial distribution for HCRU number of visits and length of stay; and GLM with Gamma distribution for costs

*Abbreviations*: *ED* Emergency department, *GLM* Generalised linear model, *HCRU* Healthcare resource utilisation, *IPTW* Inverse probability treatment weighting, *SD* Standard deviation, *SMD* Standardised mean difference (effect present if ≥ 0.1), *TNFi* tumour necrosis factor inhibitor

^a^Costs adjusted to 2022 US dollars

^b^*P*-values are from GLMs that tested for cost differences among the small fraction of members with positive costs

## Discussion

In this large, real-world study, patients with UC who were treated with a TNFi as first-line advanced therapy were 70%–80% more likely to experience inadequate response when they subsequently used a second TNFi therapy, compared with patients who switched to a non-TNFi advanced treatment. The most common individual criteria indicating inadequate response were the addition of a new conventional therapy (25%–35%) and switch to or addition of an alternative advanced therapy (15%–30%). Patients treated with a second-line TNFi therapy were 43% more likely to discontinue the drug during the first year of follow-up and more likely to have low drug adherence (32% vs. 43%), compared with patients who switched to a non-TNFi advanced therapy. Patients prescribed a second-line TNFi versus a non-TNFi exhibited comparable HCRU and cost patterns during the follow-up period. We used IPTW weighting and multivariate regression models to reduce potential confounding bias from differences in baseline patient demographic and disease characteristics between the two cohorts.

Some patients in this study may have experienced inadequate response because of drug mechanism (e.g., failure of the drug to counteract inflammatory mediators of the disease) or individual pharmacokinetics administration (e.g., subtherapeutic trough concentrations) [24]. Median time to inadequate response among patients with inadequate response was approximately 4 months, which is consistent with primary nonresponse; however, the high incidence of inadequate response in this study suggests both primary and secondary nonresponse were present, and the algorithm was not designed to differentiate between the two types.

Patients who failed the first-line TNFi therapy because of underlying biological factors (causing nonresponse to the drug mechanism of action) may have been more likely to fail a second-line TNFi [24]. Poor adherence to TNFis can result in the development of immunogenicity and consequent loss of treatment response [25]. We did not assess adherence to first-line TNFi; nonadherence and resulting immunogenicity may have disproportionately contributed to inadequate response to second-line therapy in the TNFi cohort. Better tolerance and less frequent dosing for non-TNFis (e.g., vedolizumab and ustekinumab) may have improved patients’ experience and also contributed to less switching [26, 27]. Inadequate response to treatment was also reflected in the lower persistence rate at 12 months for TNFi as second-line therapy compared with non-TNFi, which is consistent with the findings of other observational studies [25].

The treatment patterns observed in this study also reflect the changing landscape of available therapies and guidelines during the study period. In 2015, adalimumab, golimumab, infliximab, and vedolizumab became available in the US for the treatment of UC [28]. US FDA approval of tofacitinib followed in 2018 [29], ustekinumab in 2019 [30], ozanimod in 2021 [31], and upadacitinib in 2022 [32]. Further, in 2015, the then-current 2010 ACG UC practice guidelines suggested only infliximab as advanced therapy for patients refractory to conventional treatments [33]. The updated 2019 ACG guidelines recommended either a TNFi, vedolizumab, or tofacitinib, and the 2020 AGA guidelines recommended infliximab or vedolizumab, as first-line therapy for patients with more severe UC [6, 7].

Both the 2019 ACG and 2020 AGA guidelines noted the rationale for a change in drug class in selecting second-line therapy [6, 7].

Among patients in this study, 69% received a non-TNFi as second-line therapy, of whom 74% received vedolizumab and 14% ustekinumab. Agents approved later in the patient identification period were prescribed to 10% or less of patients. Some patients may have been switched to another TNFi as second-line therapy before the release of the 2019/2020 guideline recommendations to consider a different drug class after TNFi failure. Future research using most recent treatment uptake data, possibly also allowing head-to-head comparisons of newer individual agents, would be helpful to examine nuances and shifts in clinical and/or economic outcomes.

The study period encompassed the height of the COVID-19 pandemic, and nonintravenous treatments (i.e., adalimumab, ustekinumab, and tofacitinib) may have been preferentially selected to avoid exposure to medical facilities. Because intravenous treatments included both TNFis and non-TNFis, we cannot determine whether this phenomenon affected one cohort more than the other. A second-line TNFi (i.e., adalimumab) could also have been started after a first-line TNFi to change the route of administration rather than because of nonresponse, possibly accounting for some patients’ receipt of a second TNFi even after guidelines began to recommend non-TNFis as second-line advanced therapy.

While the rate of inadequate response was lower for patients who received a non-TNFi rather than a TNFi as second-line therapy (49% vs. 62%), almost half of patients receiving a non-TNFi experienced inadequate response, suggesting a need for more effective treatment options than those available in this study. Future analyses may differ because they will reflect greater use of more recently available agents, including sphingosine 1-phosphate receptor modulators (e.g., ozanimod), janus kinase (JAK) inhibitors (e.g., upadacitinib), and IL-23 inhibitors (e.g., mirikizumab) [34]. Findings of future observational studies will likely also be influenced by the increasing number of approved biosimilars, which may affect availability of agents on formulary as well as patient preferences [27].

Our analysis relied on a composite measure of inadequate response using claims data that is similar to an algorithm developed by Curtis and colleagues in the context of rheumatoid arthritis [20]. The original algorithm was benchmarked against the accepted measure of clinical effectiveness in that disease state (the disease activity score) [20]. The current algorithm was adapted for the setting of inflammatory bowel disease by Gibble and colleagues by focusing on relevant UC- and CD-specific therapeutic changes that can be observed in claims data [9]. Previous claims database studies have used a similar approach in applying composite measures of inadequate response [8, 12] or assessing individual criteria included in our inadequate response algorithm [15, 17, 35, 36]. However, clinical effectiveness measures such as rectal bleeding or mucosal healing assessed by endoscopy are not available in claims data, and validation of the algorithm against these established clinical measures is desirable.

Findings of real-world studies complement and extend the findings of randomised clinical studies. Results of our study are consistent with recent findings from network meta-analysis of clinical trials, in which ustekinumab and tofacitinib were superior to vedolizumab and adalimumab for induction of clinical remission in patients with moderate-to-severe UC and prior exposure to TNFi therapy [37]. However, our study was not designed to compare individual non-TNFi agents.

Additional real-world studies using various methodologies to investigate second-line therapy in the treatment of UC have generally found better outcomes (including drug persistence) among patients receiving vedolizumab as second-line therapy compared with TNFis [13–15]. Similarly, real-world studies that compared TNFis and non-TNFis as first-line or unspecified line of treatment for UC or CD have typically found better outcomes (e.g., adequate response, persistence, or lower dose escalation) with non-TNFi versus TNFi treatment [9, 16, 36, 38].

A recent claims analysis compared outcomes of second-line TNFi versus non-TNFi therapy in patients with several immune-mediated chronic inflammatory conditions, including UC, following initial treatment with a TNFi [17]. That study found no difference in response outcomes between second-line TNFi and non-TNFi treatment [17], a difference in findings that might be related to the different study periods, a broader group of disease states, specific agents prescribed, or differences in the patient populations (patients in the current study were younger and more likely to be male and with commercial insurance coverage, compared with the earlier study) [17]. Our findings agree with those of another recent real-world study presented at a medical meeting, in which patients with UC who were switched to a non-TNFi after initial TNFi treatment were more likely to persist on treatment and less likely to discontinue therapy than patients receiving a second TNFi [19].

Our study found overall direct healthcare costs to be slightly higher for TNFis versus non-TNFis used as second-line therapy in the treatment of UC. Specifically, UC-related inpatient costs and pharmacy costs were higher with TNFis, while outpatient costs were greater with non-TNFis. In the previously mentioned real-world analysis across multiple immune-mediated conditions, hospitalisations and ED visits were similar with TNFi versus non-TNFi second-line therapy after initial TNFi treatment; however, that study did not investigate healthcare costs [17]. Other studies have highlighted the potential costs associated with switching therapy [8] or receiving suboptimal treatment [39].

### Limitations

Claims data facilitate efficient and effective examination of healthcare outcomes, treatment patterns, resource utilisation, and costs and are collected for the purpose of payment, leading to some limitations for research use.

Any medications purchased over the counter (e.g., nonsteroidal anti-inflammatory agents) or provided as samples by a physician would not be observed in the claims data, which may underestimate use of medications. However, we expect a minimal impact on the identification of advanced therapies and a similar impact across the cohorts. Drug rebates are commonly given by manufacturers to health plans; such information was not available in the HIRD, and therefore study rebates are not reflected in the cost estimates.

We used IPTW to balance second-line TNFi and non-TNFi users on measured potential confounders, such as age, first-line UC treatments, comorbidities, and resource use. However, certain other important patient characteristics (e.g., severity of UC, member-level health-related social needs, area-level social determinants of health) were not available, and bias in the cohort comparisons due to unmeasured confounding from these variables is possible.

Considering that TNFi have been available prior to the start of our study period, and the left-censoring of patients’ observable time, some patients may have used TNFi or non-TNFi prior to the date we assigned as their 1 L treatment initiation. Our use of a 1-year “washout period” is intended to minimize such exposure misclassification.

The presence of a claim for a filled prescription does not indicate that the medication was consumed or that it was taken as prescribed. The presence of a diagnosis code on a medical claim does not guarantee the positive presence of a disease, as the diagnosis may be incorrectly coded or included as a rule-out criterion. However, we expect the impact of these limitations would not differ between the compared cohorts.

Another limitation of our study is the inability to capture clinical effectiveness measures such as endoscopic response, as these are not available in claims data. The inadequate response algorithm adapted from prior publications was validated originally for rheumatoid arthritis [20] and has yet to be separately validated for UC. Therefore, in future research there is a need to validate these criteria against clinical standards to ensure their reliability.

The non-TNFi cohort had a high proportion (> 70%) of vedolizumab users. Given that vedolizumab users may be more likely to experience inadequate response than users of IL-23 inhibitors [40–42], a different composition of non-TNFi medications may yield different outcomes.

The study results may not be fully generalisable to the overall population because patients who have commercial health insurance for at least 2 years may have different characteristics from those with shorter insurance enrolment, those with public insurance (e.g., Medicare or Medicaid), and those without health insurance. Additionally, the limited sample size may restrict the generalizability of the study findings to a broader population.

## Conclusions

Patients with UC who initially were treated with a TNFi as first-line advanced therapy and subsequently used a second TNFi had higher odds of inadequate response and were more likely to discontinue the drug compared with patients who used a non-TNFi as second-line therapy. Findings from this real-world study support guideline recommendations [6, 7] to consider a drug with a different mechanism of action after lack of response, loss of response, or intolerance of first-line TNFi therapy.

## Supplementary Information

Supplementary Material 1.

## Data Availability

The data that support the findings of this study are available from Carelon Research, but restrictions apply to the availability of these data to external sources, and therefore they are not publicly available. Data may be made available through the corresponding author upon reasonable request and with permission of Carelon Research.

## Abbreviations

ACG: American College of Gastroenterology
AGA: American Gastroenterological Association
CD: Crohn’s disease
ED: Emergency department
HCRU: Healthcare resource utilisation
HIRD: Healthcare Integrated Research Database
HR: Hazard ratio
IPTW: Inverse probability treatment weighting
PDC: Proportion of days covered
QCI: Quan-Charlson Comorbidity Index
SMD: Standardised mean difference
TNFi: Tumour necrosis factor inhibitor
UC: Ulcerative colitis
