# Supplementary material for: Real-world outcomes of tumour necrosis factor inhibitor (TNFi) cyclers versus mechanism-of-action switchers among ulcerative colitis patients who failed first-line TNFi
Source: BMC Gastroenterol. 2026 Jun 20;26:547. doi: 10.1186/s12876-026-04829-y (PMC13551955; doi:10.1186/s12876-026-04829-y)
Supplement: Supplementary file 1 [file 12876_2026_4829_MOESM1_ESM.docx]

**Supplementary Methods**

*Inadequate response individual criteria*

Individual criteria for the inadequate response algorithm were as follows:

- **Switch to or addition of non-index advanced therapies:** Patients who initiated other non-index advanced therapy medications during the 365-day follow-up period. Only on-label switch/addition was considered.
- **Addition of a new conventional therapy:** Patients who started a new conventional treatment (aminosalicylate, corticosteroid, immunomodulator, methotrexate, or leflunomide) during the 365-day follow-up period that they had not used during the baseline period.
- **Dose escalation of index medication:**
  - Dose escalation was assessed by comparing the first observed dose for the index advanced therapy after induction to all doses observed during the remainder of follow-up up to 12 months (variable period across different drugs).
    - If any daily dose of the index therapy was ≥100% higher than the first post-induction dose (i.e., twice the labelled dose), the patient was flagged as having dose escalation.
  - Daily dose was calculated as follows:
    - For outpatient prescription claims: dose = (strength x quantity dispensed)/days’ supply
    - For medical claims: dose = (strength based on Healthcare Common Procedure Coding System [HCPCS] code) x (billed units) / imputed days’ supply (based on the gaps between consecutive fills)
    - This method accounts for increased doses or shorter intervals between administration.
  - Where necessary, days’ supply was imputed as in the adherence measure (see below).
  - If both National Drug Code and HCPCS codes were found on the same claim (fill date), or within 14 days, the dose was calculated using HCPCS information only.
  - For patients who received only intravenous (IV) administrations, the number of claims was also calculated. This frequency must have been within 200% of the number expected for 1-year time period (including induction and maintenance) based on US Food and Drug Administration guidelines.
    - The following number of infusions by drug were considered as a frequency increase and were flagged:
      - Infliximab: ≥13 claims
      - Vedolizumab: ≥13 claims
      - Golimumab: ≥13 claims
    - For ustekinumab, this criterion was not applicable as only a single IV administration was used.
- **Increase in dose of oral glucocorticoids:**
  - Patients who received no prescriptions for oral glucocorticoids during the 6 months prior to the index date and received >30 days of oral glucocorticoids (as measured by days’ supply; equal to >1 fill) between index date + 90 days and index date + 365 days.
  - Patients who received prescriptions for oral glucocorticoids in the 6 months prior to the index date and received a cumulative glucocorticoid dose between index date + 184 days and index date + 365 days that was more than 120% of the cumulative dose in the 6 months prior to the index date.
    - Cumulative dose was calculated as the sum, across all fills, of strength x quantity dispensed x corticosteroid equivalent dose on each fill.
  - Both medical and pharmacy claims were assessed.
- **Use of IV glucocorticoids:** Patients who initiated IV glucocorticoid therapy in the emergency department (ED) or inpatient medical setting with a diagnosis code for UC at any position between index date + 90 days and index date + 365 days. Both medical and pharmacy claims were assessed.
- **UC-related hospitalisation or ED visit:** Presence of a UC-related inpatient visit or UC-related stand-alone ED visit between index date + 90 days and index date + 365 days. Diagnosis code for UC was considered at any position (assessed in medical claims only).
- **UC-related surgery:** Claim for a UC surgical intervention at any time between index date + 90 days and index date + 365 days in an inpatient and ED setting with a UC diagnosis code at any position (assessed in medical claims only).

*Calculation of proportion of days covered (PDC)*

PDC is calculated as the ratio of the total number of days with drug at hand to the length of the time period under investigation (from Day 0 to 365).

The total number of days with a drug at hand was calculated as follows:

For outpatient prescription claims, the days’ supply values were summed across all claims for the index drug. If days’ supply/quantity dispensed was missing, 0, or abnormal, the value was imputed based on analysis of the distribution.

For medication fills under the medical benefit, as identified through HCPCS codes, the number of days with a drug at hand was determined as the number of claims for the index drug, multiplied by the imputed days’ supply and assuming a standard regimen as per label.

For treatments under the medical benefit, the induction and maintenance frequency below was used as days’ supply. Number of fills were assigned including both medical and pharmacy fills. To account for multiple fills reported on the same claim, some maintenance dose days’ supply is not imputed as a fixed number. Such claims are calculated based on strength and quantity supply assuming normal label dose. The imputed days are based on previous experience in a similar population of interest.

Adalimumab

- Induction doses (#1 and #2) = 14 days
- Maintenance dose: Imputed days are based on the fill interval as shown below

Golimumab

- Induction dose (#1) = 14 days
- Maintenance dose: Imputed days are based on the fill interval as shown below

Infliximab

- Induction dose (#1) = 14 days
- Induction dose (#2) = 28 days
- Maintenance dose: Imputed days = 56 days

Ozanimod = 28 days (tablet)

Tofacitinib = 28 days (tablet)

Upadacitinib = 28 days (tablet)

Ustekinumab

- Induction dose (#1 IV) = 56 days
- Maintenance dose imputed days
- Ustekinumab IV: Imputed days = 56 days
- Ustekinumab SC: Imputed days are based on the fill interval as shown below

Vedolizumab

- Induction doses (#1) = 14 days
- Induction doses (#1) = 28 days
- Maintenance dose imputed days are based on the fill interval as shown below.

| **Fill interval** | **Imputed days’ supply** |
| --- | --- |
| <20 days | 14 days’ supply |
| 20-38 days | 28 days’ supply |
| 38-46 days | 42 days’ supply |
| 46-60 days | 56 days’ supply |
| >60 days | |

For interval-related days’ supply imputation: If the interval is >60 days (either there is a next fill or it is a last fill), we considered the quantity dispensed in that fill and used the following formulae. For:

- Adalimumab: Imputed days = Strength * Quantity per Fill /40*14
- Golimumab = 28 days (SC)
- Infliximab = 56 days
- Ozanimod = 28 days (Tab),
- Tofacitinib = 28 days (Tab),
- Upadacitinib = 28 days (Tab),
- Ustekinumab: Imputed days = Strength * Quantity per Fill /45*28.
- Vedolizumab: Imputed days = Strength * Quantity per Fill /150*28. If days’ supply for Generic Product Identifier/National Drug Code codes was 1 day, then imputed days = 56 days

In both cases, overlapping days’ supply across fills were subtracted from the total (with total prescription overlapping days across all fills being capped at 14 days; no cap for medical claims). If multiple claims occurred on the same date, further cleaning was applied.

*Detailed explanation of inverse probability treatment weighting (IPTW)*

Propensity scores were calculated via binomial logistic regression predicting the probability of receiving TNFi (= 1) or non-TNFi treatment (= 0) as the dependent variable. Possible covariates were selected by considering potential confounders and prior studies comparing TNFi’s and non-TNFi’s among patients with UC and included age categories, Northeast region, health plan type, payer type, index year, peripheral vascular disease, peptic ulcer disease, any malignancy, mental health conditions, anxiety, depression, obesity, arthritis, spondylitis, primary sclerosing cholangitis, first-line adalimumab, first-line golimumab, time on first-line therapy, use of immunomodulators, methotrexate, number of inpatient visits, number of outpatient visits, and total conventional and advanced therapy costs at baseline.

Overlap in the propensity score distribution and baseline covariates was balanced using IPTW; that is, samples for each cohort were weighted based on the inverse of the probability of the patient receiving their treatment.

We considered standardised mean differences (SMDs) to assess balance in means (with means considered balanced if SMD <0.1 in absolute value) and variance ratios to assess balance in variance (with variance balanced if in the range of 0.5–2), with more importance given to SMDs than variance ratios. SMD was defined as the difference in means between the TNFi/non-TNFi cohorts divided by the pooled standard deviation for continuous variables. For binary variables, the 2-sample proportion formula was used to find the difference in proportion between the TNFi/non-TNFi cohorts, which was then divided by the pooled standard deviation. For categorical variables with more than 2 levels, the SMDs were calculated using the Mahalanobis distance.

Weights were then adjusted using stabilisation (weights multiplied by the percentage of patients receiving a given treatment) and trimmed at the 1st and 99th percentiles. For assessment of balance, cost variables were log-transformed to account for skewed distribution.

Weighted cohorts were evaluated prior to between-cohort outcomes analyses. Variables that remained imbalanced after weighting were included as covariates in double-robust multivariable regression models, including the main (primary) regression analysis. All variables for the main regression model as well as the sensitivity analyses are shown in Supplementary Table 2.

**Supplementary Tables**

Supplementary Table 1 Patient selection

| **Criterion** | **Patients (%)** |
| --- | --- |
| Patients with ≥1 medical or pharmacy claim for a TNFi or non-TNFi advanced agent approved for UC during the patient identification period (1/1/2015 to 6/30/2022)^a^ | 187,722 |
| Continuous enrolment for 1 year before first-line claim date | 99,684 (53) |
| No claim for a TNFi or non-TNFi advanced agent approved for UC in 1 year before first-line claim date | 74,417 (75) |
| No claim for a non-TNFi advanced agent approved for UC on first-line claim date | 57,420 (77) |
| No claim for >1 TNFi advanced agent approved for UC on first-line claim date | 57,419 (100) |
| ≥1 medical or pharmacy claim for a non-first-line TNFi approved for UC or non-TNFi advanced agent approved for UC, between the end date of first-line therapy and end of study period (1/1/2014 to 6/30/2023)^b^ | 12,626 (22) |
| Continuous eligibility for 1 year after index date | 9,310 (74) |
| ≥2 claims for UC at least 30 days apart during the study period | 2,599 (28) |
| ≥1 claim for UC from start of study period to first-line claim date (inclusive) | 2,399 (92) |
| ≥1 claim for UC from 183 days prior to the index date to index date (inclusive) | 2,073 (86) |
| Exclude patients with total colectomy pre-index | 2,037 (98) |
| Aged ≥18 years on the index date | 1,882 (92) |
| Continuous eligibility from first-line claim date to index date + 12 months | 1,807 (96) |
| Only 1 claim for TNFi or non-TNFi advanced agent approved for UC on index date | 1,806 (100) |
| Exclude patients with CD diagnosis on or after first-line claim date | 1,074 (59) |
| Exclude patients with ≥2 claims for other autoimmune conditions (ankylosing spondylitis, hidradenitis suppurativa, juvenile idiopathic arthritis, multiple sclerosis, psoriasis, psoriatic arthritis, rheumatoid arthritis, uveitis) 30 days apart from start of study period to first-line claim date | 933 (87) |
| Second-line index drug: non-TNFi | 646 (69) |
| Second-line index drug: TNFi | 287 (31) |

Patients in each row are selected from the patients in the previous row. Percentage refers to proportion of patients in the previous row.

Abbreviations: CD, Crohn's disease; N/A, not applicable; TNFi, tumour necrosis factor inhibitor; UC, ulcerative colitis.

^a^Date of first claim is identified as the first-line claim date.

^b^Date of first such claim (for the second-line index drug) is identified as the index date.

Supplementary Table 2 Summary of variables included in the main (primary) regression and sensitivity analysis models

| **Variable Name** | **Main regression^a^** | **Sensitivity analysis 1^b^** | **Sensitivity analysis 2^c^** | **Sensitivity analysis 3^d^** |
| --- | --- | --- | --- | --- |
| Baseline all-cause length of stay | No | No | Yes | No |
| Baseline all-cause allowed total medical costs | No | No | Yes | No |
| Baseline all-cause allowed inpatient costs | No | No | Yes | No |
| Baseline all-cause allowed ED costs | No | No | Yes | No |
| Baseline all-cause allowed total costs | No | No | Yes | No |
| Baseline UC length of stay | Yes | Yes | Yes | No |
| Baseline UC ED visit count | No | No | Yes | No |
| Baseline UC allowed inpatient costs | No | No | Yes | No |
| Baseline UC allowed ED costs | No | No | Yes | No |
| Baseline UC allowed medical costs | No | No | Yes | No |
| Baseline UC allowed conventional and advanced therapy costs medical cost without UC | No | No | Yes | No |
| Baseline UC allowed total costs | No | No | Yes | No |
| Baseline UC allowed medical costs minus Ulcerative colitis advanced conventional costs | No | No | Yes | No |
| Index age ≥75 years | Yes | No | No | No |
| Region unknown | Yes | Yes | Yes | No |
| Baseline QCI score | Yes | Yes | Yes | No |
| Baseline QCI: cerebrovascular disease | No^e^ | No^e^ | No^e^ | No |
| Baseline QCI: dementia | No^e^ | No^e^ | No^e^ | No |
| Baseline QCI: moderate/severe liver disease | No^e^ | No^e^ | No^e^ | No |
| Baseline QCI: diabetes without complications | Yes | Yes | Yes | No |
| Baseline QCI: diabetes with CC | Yes | Yes | Yes | No |
| Baseline QCI: metastatic solid tumour | Yes | Yes | Yes | No |
| Baseline Crohn’s disease | Yes | Yes | Yes | No |
| Baseline spondylitis | Yes | Yes | Yes | No |
| Baseline pyoderma gangrenosum | Yes | Yes | Yes | No |
| Baseline autoimmune hepatitis | Yes | Yes | Yes | No |
| Baseline mesalamine | Yes | Yes | Yes | No |
| Baseline tacrolimus | No^e^ | No^e^ | No^e^ | No |
| Sex | Yes | Yes | Yes | No |
| Propensity score model variables |  |  |  |  |
| Age categories | No | Yes | Yes | No |
| Northeast region | No | Yes | Yes | No |
| Health plan type | No | Yes | Yes | No |
| Payer type | No | Yes | Yes | No |
| Index year | No | Yes | Yes | No |
| Peripheral vascular disease | No | No^e^ | No^e^ | No |
| Peptic ulcer disease | No | No^e^ | No^e^ | No |
| Any malignancy | No | Yes | Yes | No |
| Mental health conditions | No | Yes | Yes | No |
| Anxiety | No | Yes | Yes | No |
| Depression | No | Yes | Yes | No |
| Obesity | No | Yes | Yes | No |
| Arthritis | No | No^e^ | No^e^ | No |
| Spondylitis | No | No^e^ | No^e^ | No |
| Primary sclerosing cholangitis | No | No^e^ | No^e^ | No |
| First-line therapy: adalimumab | No | Yes | Yes | No |
| First-line therapy: golimumab | No | Yes | Yes | No |
| Time on first-line therapy, days | No | Yes | Yes | No |
| Immunomodulators | No | Yes | Yes | No |
| Methotrexate | No | Yes | Yes | No |
| Number of inpatient admissions | No | Yes | yes | No |
| Number of outpatient visits | No | Yes | yes | No |
| Conventional and advanced therapy costs, total | No | Yes | yes | No |

^a^Weighted model with covariates: all baseline-imbalanced variables with IPTW (excluding those balanced after log transform).

^b^Weighted model with covariates: all baseline-imbalanced variables (excluding those balanced after log transform); all variables used for generating the propensity scores.

^c^Weighted model with covariates: all baseline-imbalanced variables; all variables used for generating the propensity scores; log-transform applied.

^d^Weighted model no covariates.

^e^Removed from primary model and sensitivity analyses due to low frequency.

Abbreviations: ED, emergency department; IPTW, inverse probability treatment weighting; QCI, Quan-Charlson Comorbidity Index; UC, ulcerative colitis.

Supplementary Table 3 Summary of sensitivity analyses for odds of the inadequate response composite endpoint over 12 months after the index date (*n* = 921)

| **Model** | **Estimate (SE)** | **Odds ratio (95% CI)** | ***p*-value** |
| --- | --- | --- | --- |
| Main regression model | 0.566 (0.150) | 1.76 (1.31–2.37) | <0.0001 |
| Sensitivity analysis model 1 | 0.593 (0.157) | 1.81 (1.33–2.46) | <0.001 |
| Sensitivity analysis model 2 | 0.590 (0.160) | 1.80 (1.32–2.47) | <0.001 |
| Sensitivity analysis model 3 | 0.511 (0.146) | 1.67 (1.25–2.22) | 0.001 |

Based on logistic regression models.

Abbreviations: CI, confidence interval; SE, standard error.

Supplementary Table 4 Ulcerative colitis–related HCRU and costs over 12 months after the index date

|  | **TNFi** **(*n* = 281)** | **Non-TNFi** **(*n* = 640)** | ***p*-value** | **SMD** |
| --- | --- | --- | --- | --- |
| HCRU |  |  |  |  |
| Inpatient |  |  |  |  |
| ≥1 visit, *n* (%) | 49 (18) | 90 (14) | 0.171 | 0.10 |
| Visits, mean (SD) | 0.3 (0.9) | 0.2 (0.7) | 0.103 | 0.12 |
| Length of stay among those with ≥1 visit, days, mean (SD) | 8.1 (10.6) | 7.5 (8) | 0.605 | 0.06 |
| ED |  |  |  |  |
| ≥1 visit, *n* (%) | 17 (6) | 62 (10) | 0.057 | 0.14 |
| Visits, mean (SD) | 0.1 (0.4) | 0.1 (0.5) | 0.205 | 0.11 |
| Outpatient |  |  |  |  |
| ≥1 visit, *n* (%) | 278 (99) | 638 (100) | 0.173 | 0.09 |
| Visits, mean (SD) | 16.2 (11.7) | 17 (10) | 0.160 | 0.07 |
| Costs, $, mean (SD) [median]^a^ |  |  |  |  |
| Total medical | 60,252 (16,1568) [23,450] | 56,044 (65,661) [53,552] | 0.429 | 0.03 |
| Inpatient | 23,228 (146,804) [0] | 7,259 (26,754) [0] | <0.001^b^ | 0.15 |
| ED | 363 (1,598) [0] | 628 (2,617) [0] | 0.824^b^ | 0.12 |
| Outpatient | 36,660 (60,356) [19,749] | 48,157 (57,129) [49,331] | 0.002 | 0.20 |
| Therapy costs^c^ covered under pharmacy benefit | 46,268 (45,592) [33,582] | 34,311 (54,266) [5,903] | 0.046 | 0.24 |
| Therapy costs^c^ covered under medical benefit^d^ | 436 (2,773) [0] | 360 (3,666) [0] | 0.835^b^ | 0.02 |
| Total | 106,956 (158,385) [87,067] | 90,714 (71,006) [70,745] | <0.001 | 0.13 |

IPTW-weighted cohort. The mean numbers of visits/fills and mean costs are calculated out of the total number of patients in each cohort. *P*-values were calculated as follows: Chi^2^ tests for HCRU percentage comparisons; GLM with negative binomial distribution for HCRU number of visits and length of stay; and GLM with Gamma distribution for costs.

Abbreviations: ED, emergency department; GLM, generalised linear model; HCRU, healthcare resource utilisation; IPTW, inverse probability treatment weighting; SD, standard deviation; SMD, standardised mean difference (effect present if ≥0.1); TNFi, tumour necrosis factor inhibitor.

^a^Costs adjusted to 2022 US dollars.

^b^ P-values are from GLMs that tested for cost differences among the small fraction of members with positive costs.

^c^Conventional therapies (aminosalicylates, corticosteroids, immunomodulators, methotrexate, and leflunomide) and advanced treatments (adalimumab, golimumab, infliximab, ozanimod, tofacitinib, upadacitinib, ustekinumab, and vedolizumab).

^d^Costs not captured under “Total medical costs”.

Supplementary Figure

Supplementary Fig. 1 Illustration of the study time periods. For example, the study period was defined first, after which the first-line intake (patient identification) period was defined. The numbers in the figure denote steps in operationalising the study design.

Abbreviations: 1L, first-line; 2L, second line; TNFi, tumour necrosis factor inhibitor; UC, ulcerative colitis.
